# Emergency general surgery, malnutrition and outcomes in critical illness

**DOI:** 10.1186/2197-425X-3-S1-A447

**Published:** 2015-10-01

**Authors:** JM Havens, O Olufajo, KM Mogensen, JD Rawn, A Salim, KB Christopher

**Affiliations:** 1 Brigham and Women's Hospital, Division of Trauma, Burn, and Surgical Critical Care, Boston, MA USA; 2 Department of Nutrition, Brigham and Women's Hospital, Boston, MA USA; 3 Division of Cardiac Surgery, Brigham and Women's Hospital, Boston, MA USA; 4 Renal Division, Brigham and Women's Hospital, Boston, MA USA

## Introduction

Emergency General Surgery (EGS) is a rapidly evolving but understudied branch of Acute Care Surgery. Limited information exists regarding the contribution of malnutrition to the outcome of critically ill patients who undergo EGS.

## Objectives

We hypothesized that malnutrition would be associated with increased risk of 90 day all cause mortality following ICU admission.

## Methods

We performed a single center observational study of patients treated in medical and surgical ICUs in Boston. We studied 1,361 patients age ≥ 18 years, who received critical care and an EGS associated procedure between 1997 and 2011. The exposure of interest, malnutrition, was determined in patients at risk for malnutrition via a Registered Dietitian formal assessment within 48 hours of ICU admission and categorized as non-specific malnutrition, protein-energy malnutrition, or without malnutrition. Nutrition data was determined via anthropometric measurements, clinical signs of malnutrition, malnutrition risk factors, and metabolic stress. Energy and protein delivery goals were determined by the Registered Dietitian. The primary outcome was all cause 90 day mortality determined by the US Social Security Death Master File. Adjusted odds ratios were estimated by multivariable logistic regression models.

## Results

The cohort was 58% male, 74% white and had a mean age of 60.1 years. 26% of the cohort had sepsis, 14% had acute kidney injury and 33% had non-cardiac acute respiratory failure. 17% of the cohort were readmitted to the Brigham and Women’s Hospital or Massachusetts General Hospital within 30 days of discharge. 60% had non-specific malnutrition, 8% had protein-energy malnutrition, and 32% without malnutrition. The 30, 90 and 365-day mortality was 9.1 and 17.9 and 29.6%. In an logistic regression model adjusted for gender and an ICU risk prediction score (inclusive of age, gender, race, Deyo-Charlson index, acute organ failure, and sepsis), patients with non-specific malnutrition have a 1.6 fold increased odds of 90-day mortality [adjusted OR 1.56 (95%CI 1.12-2.18), *P* = 0.009] and in patients with protein-energy malnutrition a 3.0 fold increased odds of 90-day mortality [adjusted OR 2.96 (95%CI 1.78-4.92), *P* < 0.001] compared to patients without malnutrition. Further, in survivors of hospitalization (n = 1,223) patients with protein-energy malnutrition at ICU admission have a 2.8 fold increased odds of 90-day post discharge mortality [adjusted OR 2.77 (95%CI 1.49-5.17), *P* = 0.001] compared to patients without malnutrition. In a subset of patients with detailed follow up nutrient intake data (n = 533), the mean (SD) percent of goal energy and protein delivered was 48.3 (31) and 44.7 (30) during the seven days following EGS.

## Conclusions

In critically ill patients who undergo EGS, malnutrition at ICU admission is predictive of adverse outcomes. In survivors of hospitalization, malnutrition at ICU admission is associated with increases in mortality following discharge.

